# *β*-elimination of hyaluronate by red king crab hyaluronidase

**DOI:** 10.1038/s41598-021-01890-3

**Published:** 2021-11-19

**Authors:** Dmitrii Sliadovskii, Tatyana Ponomareva, Maxim Molchanov, Irina Pozdnyakova-Filatova, Maria Timchenko, Victor Marchenkov, Oleg Gusev, Evgeny Sogorin

**Affiliations:** 1 Federal Research Center “Pushchino Scientific Center for Biological Research of the RAS”, Pushchino, Russia 142290; 2 Institute of Theoretical and Experimental Biophysics of the RAS, Pushchino, Russia 142290; 3 Federal Research Center “Pushchino Scientific Center for Biological Research of the RAS”, G.K. Skryabin Institute of Biochemistry and Physiology of Microorganisms, Pushchino, Russia 142290; 4 Institute of Protein Research RAS, Pushchino, Russia 142290; 5 Regulatory Genomics Research Center, Institute of Fundamental Medicine and Biology, Kazan Federal University, Kazan, Russia 420012; 6 Graduate School of Medicine, Juntendo University, Tokyo , 113-8421 Japan

**Keywords:** Biocatalysis, Enzyme mechanisms, Enzymes, Biochemistry, Carbohydrates, Polysaccharides, Biotechnology, Animal biotechnology, Computational biology and bioinformatics, Functional clustering, Protein analysis, Sequence annotation

## Abstract

Crustacean hyaluronidases are poorly understood both in terms of their enzymatic properties and in terms of their structural features. In this work, we show that the hepatopancreas homogenate of the red king crab has a hyaluronidase activity that is an order of magnitude higher than its commercial counterpart. Zymography revealed that the molecular weight of a protein with hyalorunidase activity is 40–50 kDa. Analysis of the hepatopancreas transcriptome and results of cloning and sequencing of cDNA revealed a hyaluronidase sequence with an expected molecular weight of 42.5 kDa. Further analysis showed that hyaluronat enzymatic cleavage follows the $\beta$-elimination mechanism, which is well known for bacterial hyaluronidases. The results of ion-exchange chromatography showed that the final product of hyaluronate degradation is unsaturated tetrasaccharide. Thus, we identified a new hyaluronidase of higher eukaryotes, which is not integrated into the modern classification of hyaluronidases.

## Introduction

**Figure 1:**
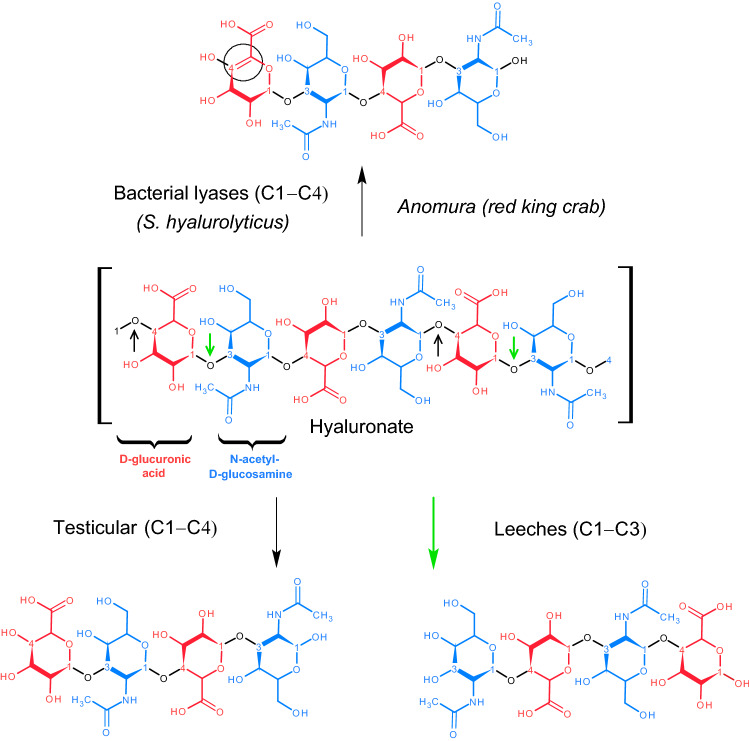
The structure of HA and the products of the hydrolysis reaction by different classes of hyaluronidases. Small arrows in the picture of the HA structure indicate the places of the bond break (green arrows—leeches, black—the rest), a circle marks the double bond between C-4 and C-5 glucuronic acid atoms, which is formed during the $\beta$-elimination of HA.

Hyaluronidases are a group of enzymes that can break down hyaluronate (HA) (Fig. 1). Most of them are also able to break down chondroitin and its derivatives^1^. Hyaluronidases are divided into three classes depending on the characteristics of their enzymatic activity^2,3^. HA-4-glycanohydrolases cleave the $\beta$-(1$\rightarrow$4)-glycosidic bonds of the HA chain to form N-acetyl-D-glucosamine at the reduced end of the oligosaccharides including the hyaluronidases of mammals and some insects. HA-3-glucanohydrolases of leech hyaluronidases hydrolyze $\beta$-(1$\rightarrow$3)-glycosidic bonds. The reaction products contain N-acetyl-D-glucosamine at the non-reduced end and D-glucuronic acid at the reduced end of the oligosaccharides. The third group—bacterial hyaluronidase (HA lyases)—cleaves $\beta$-(1$\rightarrow$4)-glycosidic bonds by the mechanism of $\beta$-elimination to form a double bond in D-glucuronic acid^4^. Interestingly, hyaluronidase from *Streptomyces hyalurolyticus* is capable of cleaving HA at $\beta$-(1$\rightarrow$3)-glycosidic bonds, producing even and odd-numbered oligosaccharides as a minor product^5,6^. Odd-numbered oligosaccharides are formed by the cleavage of N-acetyl-D-glucosamine at the non-reduced end and its conversion to $\Delta$HexNAc. In this case, the free $\Delta$HexNAc, as well as formed D-glucuronic acid at the non-reduced end, contain a double bond^5^.

$\beta$-elimination of HA is usually identified only with bacterial hyaluronidases. However, the same type of hydrolysis was recently shown for the yeast *Cryptococcus laurentii*^7^ and for the tinder fungus *Fistulina hepatica*^8^ (Table 1).

To date, hyaluronidases have been widely used in various fields of medicine: in local anesthesia^9,10^, in ophthalmology^11,12^, in dermatosurgery^13^. According to market research (2018), about 65% of the medications are animal-derived hyaluronidases, the rest are recombinant forms^14^. A significant part of the preparations are of animal origin, namely homogenates of testicular tissues (for example, Lydazum, Liporaza, Vitrase, Amphadase and others).

The popularity of medical preparations obtained on the basis of testicular tissues of farm animals can be explained by the low cost of raw materials and the simplicity of obtaining the final drug. For example, the drug Lydazum is obtained as follows: tissues shredding, extraction with acetic acid, centrifugation, concentration of the supernatant, ultrafiltration through membranes with a pore size of 50 kDa^15^. The resulting homogenate contains a heterogeneous mixture of proteins (Supplementary Fig. 1).

The hyaluronidases of humans, some animals, and bacteria are well described both in terms of their biochemical properties and structural features^1,16,17^. However, crustacean hyaluronidases are poorly described. The hyaluronidase of langust *Nephrops norvegicus* is an enzyme of 320 kDa with an optimum activity of 45 $^\circ$C and a pH of 5.4. The reaction mechanism of HA hydrolysis by langoustine hyaluronidase is still unknown. The HA-3-glycanohydrolase of antarctic krill *Euphausia superba* has a molecular weight of 80 kDa characterized by an optimal enzymatic activity of 37 $^\circ$C and a pH of 5.3^18^. To date, the amino acid sequences of langoustine and krill hyaluronidases are not known. Hyaluronidase protein sequences of Decapod order (Decapoda) can be found on UniProt database, among which are: white-legged shrimp (*Litopenaeus vannamei*, UniProt A0A423SH46) and the orange mud crab (*Scylla olivacea*, two UniProt A0A0P4VVV1 and A0A0N7ZAX3); as well as two representatives of the order of Equipods (Isopoda order): woodlouse *Armadillidium vulgare* (UniProt A0A444ST78) and *Armadillidium nasatum* (UniProt A0A5N5TJL6). However, the biochemical properties of these hyaluronidases have not been studied yet.

Recently we showed that hepatopancreas homogenate of the red king crab can induce the cleavage of the HA in cosmetic filler^19^. In this work we identified the amino acid sequence of the red king crab hyaluronidase, which differs significantly in terms of the amino acid sequences of known hyaluronidases. We shown that the red king crab hyaluronidase cleaves $\beta$-(1$\rightarrow$4)-glycosidic bonds by the mechanism of $\beta$-elimination. The results of ion-exchange chromatography showed that the final product of HA degradation is unsaturated tetrasaccharide. The rate of HA cleavage is an order of magnitude higher than the rate of hydrolysis by a commercial analog (Lydazum) when normalized to the total protein in the studied preparations of hyaluronidase. Since red king crab catch amounts to large volumes in different countries generating hepatopancreas as a waste product of crab processing it is safe to assume that it generates enough of a raw material for production of a new potential medical drug hyaluronidase on industrial scale^20^.

**Table 1:** Some known hyaluronidases.

Hyaluronidase	Opt. $^\circ$C and pH	Substrate specificity	Additional information
Testicular	?, 5.0^21^	HA, chondroitin sulfate^22^	Hydrolysis at the $\beta$-1,4-glycosidic bond. Final product is tetrasaccharide^23^. Capable of transglycosylation^21,24^
Leeches	45 and 6.5^25^	HA, does not hydrolyze chondroitin sulfate A and C^26^, and also does not hydrolyze chitin and heparin^25^	Hydrolysis at the $\beta$-1,3-glycosidic bond, the final product—hexa- and tetrasaccharide—contains glucuronic acid at the reduced end^25–27^
*Streptomyces hyalurolyticus*	60 and 5.0^23^	HA, does not hydrolyze chondroitin sulfate, and also does not hydrolyze chitin, heparin, and keratan sulfate^23^	The reaction of $\beta$-elimination at the $\beta$-1,4-glycosidic bond, hexa- and tetrasaccharide are the final products of the endolytic activity of hydrolysis^23,28,29^
*Bacillus sp. A50*^30^	44 and 6.5	HA, chondroitin sulfate A, C, D	The reaction of $\beta$-elimination at the $\beta$-1,4-glycosidic bond, the final product is a dimer
*Cryptococcus laurentii*^7^ (yeast)	37 and 6.0	HA, ?	The reaction of $\beta$-elimination at the $\beta$-1,4-glycosidic bond, the final product is a dimer
*Fistulina hepatica*^8^ (filamentous fungi)	20 and 4.0	HA, ?	The reaction of $\beta$-elimination at the $\beta$-1,4-glycosidic bond, the final product is tetrasaccharide

## Results

### Transcriptome analysis of red king crab hepatopancreas, cloning and sequencing of cDNA encoding the mature form of hyaluronidase

First *de-novo* transcriptome assembly allowed us to create a database consisting of 101,101 contigues covering a total transcriptome of the red king crab. The length of the transcripts varied from 200 bp to 20,595 bp, with an average length of 720 bp. Next, from the obtained transcripts, we created a local BLAST database and identified a potential gene for the red king crab hyaluronidase based on the similarity with the known arthropod hyaluronidase genes. The transcript containing the coding part of hyaluronidase consisted of 1628 bp with an open reading frame of 1119 bp encoding 372 amino acid protein. The primary amino acid sequence of the identified gene was closest to shrimp hyaluronidase *Penaeus vannamei*: (Score = 375.9 bits (964), Expect = 3E-125, Identities = 172/313 (54%), Positives = 224/313 (72%), Gaps = 3/313 (1%) (BLAST database).

To clone cDNA hyaluronidase, a total red king crab hepatopancreas (HPC) RNA preparation was obtained. Single-stranded DNA of all polyadenylated mRNAs was produced using oligo (dT)18-primer. After that, PCR was performed using primer oligonucleotides complementary to the hyaluronidase gene sequence identified in red king crab transcriptome analysis. The exception is the position of the amino acid Tyr193, in the codon of which there was a replacement: UAC (cloning)$\rightarrow$UAU (transcriptome). In the future the resulting DNA sequence will be used to produce an expression system plasmid construct.

### Search for homologues of the identified amino acid sequence of red king crab hyaluronidase

The SwissProt database contains the amino acid sequences of the following enzymes that can use HA as a substrate (date of reference to the database is 06.06.2021):

- EC 3.2.1.35 (catalyzes the cleavage of the $\beta$-(1$\rightarrow$4) bond)—61 representatives, of which 2 are found in bacteriophages attacking the bacteria of Streptococcus genus (P15316 and Q54699), and 59 representatives are found in organisms of the kingdom Animalia (classes Insecta, Arachnida, Reptilia, Mammalia, as well as Gastropoda (genus Conus)).
- EC 3.2.1.36 (catalyzes the cleavage of the $\beta$-(1$\rightarrow$3) bond)—1 representative, found in *Hirudo nipponia* (Korean blood-sucking leech) (X4Y2L4).
- EC 4.2.2.1 (catalyzes the cleavage of the $\beta$-(1$\rightarrow$4) bond, $\beta$-elimination)—9 representatives (7 of them are found in bacteria and 2 in *Homo sapiens*), 3 of them (Q12794 (*Homo sapiens*), Q12891 (*Homo sapiens*) and P26831 (*Clostridium perfringens*)) also apply to EC 4.2.2.1 and EC 3.2.1.35.

**Figure 2:**
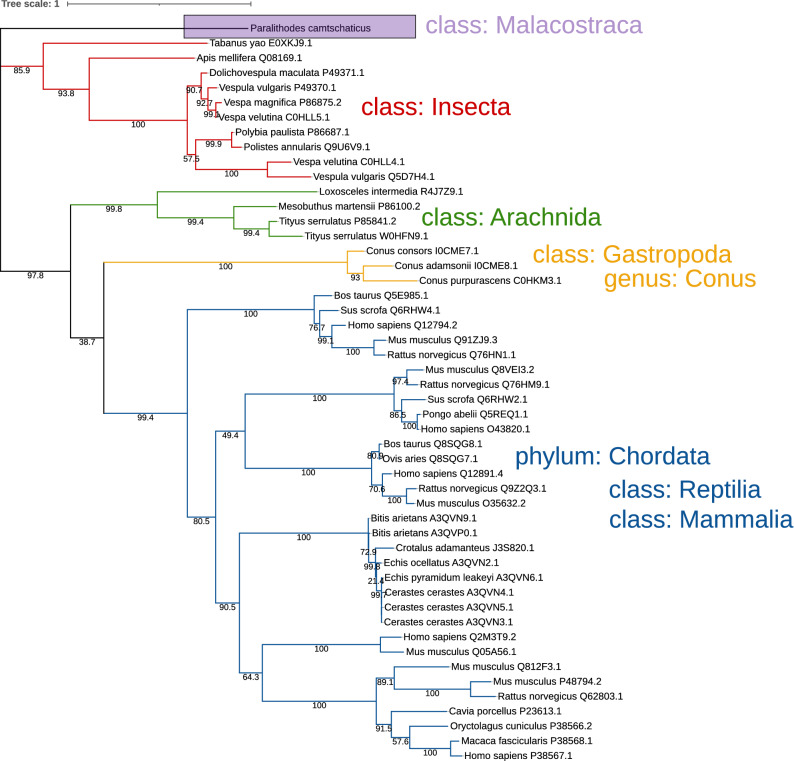
Phylogenetic tree of the homologues of the red king crab hyaluronidase, constructed on the basis of the Swiss-Prot database. Homologues were searched in the Swiss-Prot database using BLASTP with default parameters. A phylogenetic tree was constructed from MUSCLE-aligned sequences of hyaluronidases with the help of IQ-TREE^31^. For visualization, iTOL was used^32^, bootstrap values are indicated at the branch points.

The red king crab hyaluronidase has a length of 372 amino acid residues (Supplementary Fig. 2) corresponding to the molecular weight of 42.5 kDa, and is the first hyaluronidase described for the Anomura Infraorder. Among the orthologs detected by BLASTP in the SwissProt database there are hyaluronidases of the class 3.2.1.35 (including Q12794 (*Homo sapiens*), and Q12891 (*Homo sapiens*)). At the same time, the hyaluronidase of the red king crab is not included in any of the clades formed by known representatives of this class (Fig. 2).

### Cleavage of HA at the $\beta$-(1$\rightarrow$4)-glycosidic bond in the presence of HPC homogenate

The enzymatic activity of the red king crab hypatopancreas homogenate was determined by using a colorimetric method based on the Morgan-Elson reaction^33,34^, which leads to a chromogen formation (a color reaction product). N-acetyl-D-glucosamine heated under alkaline conditions forms furan derivatives that react with *p*-dimethylaminobenzaldehyde to form a red dye which is detected with spectrophotometry. Intense color is associated with high production of N-acetyl-D-glucosamine at the reduced end of the chain.

**Figure 3:**
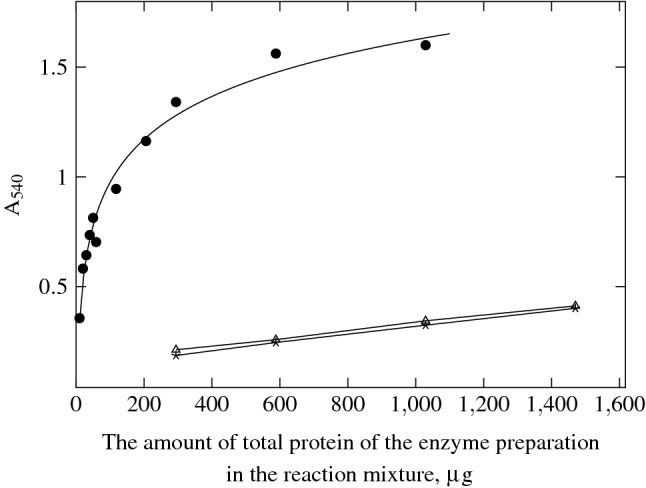
The accumulation of the products of the HA cleavage reaction using the HPC homogenate and the commercial preparation Lydazum depending on the amount of the total protein of the preparation in the reaction mixture, incubation at 38 ${}^\circ$C, 20 min, in an acetate buffer (pH 5.62): HPC homogenate,  preparation Lydazum,  preparation Lydazum (repeat).

The commercial drug Lydazum was used as a standard control^35^ for hyaluronidase activity measurement. The cleavage of HA with HPC homogenate was performed under conditions close to optimal (Supplementary Fig. 3). Remarkably the hyaluronidase of the HPC homogenate exceeds the commercial drug activity by order of magnitude when normalized to the drug’s total protein amount per reaction mixture (Fig. 3). Colored product detection indicates the cleavage of HA at the $\beta$-1,4-glycosidic chain bond in the presence of the HPC homogenate enzyme. The colored product is not observed in the case of leech^25^ or antarctic krill *Euphausia superba* hyaluronidases^18^ because they hydrolyze HA at $\beta$-(1$\rightarrow$3)-glycosidic bond to produce N-acetyl-D-glucosamine at the non-reduced end of the reaction products, which is not amenable to the Morgan-Elson reaction. The specific activity of the HPC homogenate was 2,100 units of hyaluronidase activity per 1 mg of the total protein (drug Lydazum had 87 units of hyaluronidase activity per 1 mg of the total protein). The titration curve of the HPC homogenate activity units almost precisely overlapped the commercial drug *S. hyalurolyticus* lyase (Sigma H1136) (Supplementary Fig. 4).

### Zymography

**Figure 4:**
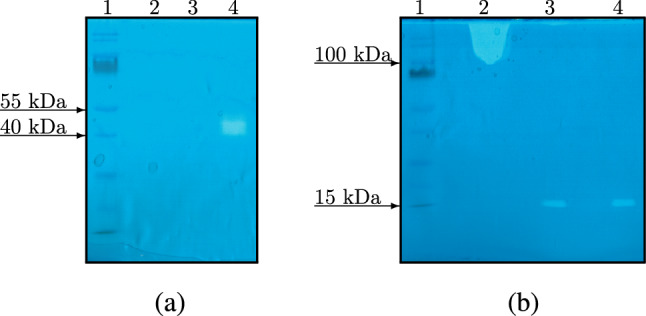
Zymogram in polyacrylamide gel containing HA as a substrate: (**a**)—HPC homogenate (1—protein marker, 2—homogenate after heating in the loading buffer, 3—homogenate after heating in the loading buffer without DTT, 4—homogenate in buffer with DTT without warming up), (**b**)—*S. hyalurolyticus* lyase (1—protein marker, 2—after warming up in buffer without DTT, 3—after warming up in buffer with DTT, 4—hyaluronidase in buffer with DTT without warming up). Protein marker PageRuler Prestained Protein Ladder: 180, 130, 100, 70, 55, 40, 35, 25, 15, 10 kDa. The samples were being heated in the loading buffer for 5 min at 95 ${}^\circ$C.

Zymography of the HPC homogenate revealed the protein with hyaluronidase activity is roughly 40–50 kDa relative to the molecular marker used in electrophoresis (Fig. 4(a), full–length gel is presented in Supplementary Fig. 5(a)). In addition, heating of the HPC homogenate preparation in the sample buffer (with or without DTT) leads to an irreversible loss of hyaluronidase activity. This is not observed in the case of activity detection by zymography of hyaluronidase from actinobacterium *Streptomyces hyalurolyticus*—the enzyme retains its activity after heating in the buffer, but the presence of DTT in it prevents the formation of aggregates after heating (Fig. 4(b), full–length gel is presented in Supplementary Fig. 5(b)). According to the protein marker, the molecular weight of *S. hyalurolyticus* lyase is in the range of 15 to 25 kDa, which is close to *S. koganeiensis* lyase (21.6 kDa)^36,37^. It should be noted that the molecular weight of *S. hyalurolyticus* lyase declared by the manufacturer is 91 kDa, which was determined by the results of gel filtration. Such a large difference can be explained by the oligomer formation of *S. hyalurolyticus* lyase under gel-filtration conditions.

Temperature dependent loss of the hyaluronidase activity may be associated with irreversible thermal denaturation of the hyaluronidase, or with the activation of proteolytic enzymes of the HPC homogenate at high temperatures during heating. In this case, it should be assumed that the presence of a detergent at a concentration of 0.2% (SDS) in the buffer did not radically affect the activity of some proteolytic enzyme of the HPC homogenate. Notably, hyaluronidase activity of the HPC homogenate in the substrate gel produced an extended band. This is probably due to the influence of post-translational modifications (glycosylation reduces the electrophoretic mobility of the protein). It was previously shown that the deglycosylation of the sperm membrane hyaluronidase (PH-20 protein) leads to an increase in its electrophoretic mobility in the gel^38^. Mobility of recombinant bee hyaluronidase of *Apis mellifera* isolated from *E. coli* (i.e. without glycosylation) also differed in mobility from the native protein^39^. Thus, the observed wide band of activity in the gel might be due to the glycosylation of hyaluronidase molecules in the homogenate, which leads to a spread in electrophoretic mobility.

### Formation of a double bond during the *β*-elimination of HA in the presence of HPC homogenate

**Figure 5:**
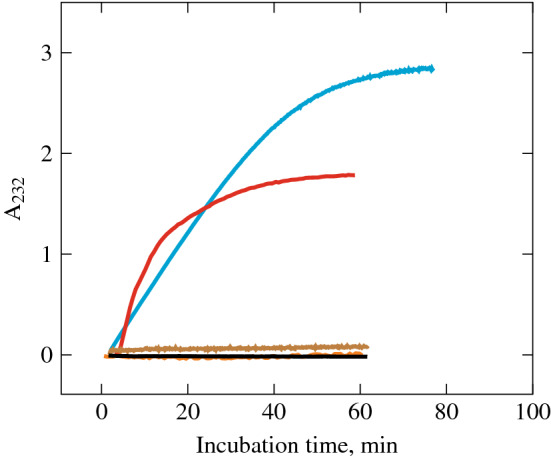
*In situ*-monitoring of the double bond formation during the $\beta$-elimination reaction of HA (2 mg/ml HA in the phosphate buffer, pH 6, 37 ${}^\circ$C):  *S. hyalurolyticus* lyase,  HPC homogenate,  «Lydazum»,  HPC homogenate without HA,  leech head homogenate.

The formation of a double bond between C-4 and C-5 glucuronic acid atoms during the $\beta$-elimination of HA makes it possible to detect the *in situ* reaction kinetics by measuring the UV absorption in reaction mixture (Fig. 5). As a positive control, the *S. hyalurolyticus* lyase was used causing an increase of a 232 nm UV absorption in the reaction mixture indicating the formation of a double bond during the cleavage of HA by the $\beta$-elimination reaction. As a negative control, the preparation of testicular hyaluronidase (Lydazum) and leech head homogenate were used. As expected, both of these hyaluronidases did not lead to an increase in the UV absorption of the reaction mixture, despite their hydrolysis of HA, which is detected by other methods under the same conditions (Supplementary Fig. 6). Incubation of HPC homogenate in a phosphate buffer without HA in the mixture also did not lead to an increase in absorption at a wavelength of 232 nm. Immediately after the addition of the HPC homogenate to the HA solution, an increase in the absorption of UV radiation was observed, which indicates the cleavage of HA by the homogenate enzymes by the $\beta$-elimination mechanism, as in the case of bacterial lyase from our positive control.

### Analysis of HA hydrolysis products by $^1$H-NMR spectroscopy

NMR spectroscopy has previously been used to analyze reaction products and study the kinetics of the HA cleavage reaction^19,29,40–42^. As we demonstrated earlier studying the HA of a cosmetic filler, NMR analysis of the reaction mixture can be successfully used to evaluate the kinetics of HA cleavage^19^. As shown, the cleavage of HA by the enzymes of the HPC homogenate leads to an increase in the signals of the protons of the acetyl group of N-acetyl-D-glucosamine in the HA: two peaks in the range 2.08–2.02 ppm. One of them corresponds to the proton signal of the N-acetyl-D-glucosamine acetyl group inside the HA chain (“inside protons”, peak 2.032 ppm), and the other one emanates from the N-acetyl-D-glucosamine acetyl group of the HA fragment ends during the cleavage process (“end protons”, 2.074 ppm).

**Figure 6:**
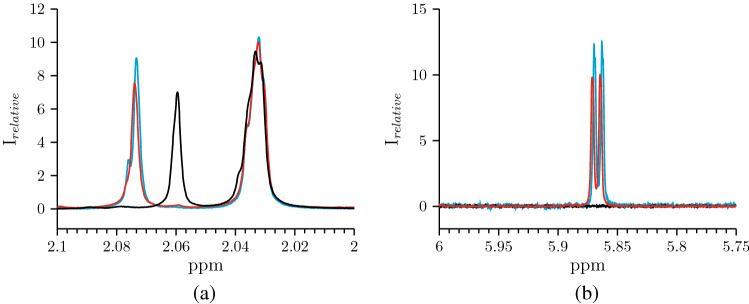
$^1$H-NMR spectra of the reaction mixtures containing HA after treatment by:  *S. hyalurolyticus* lyase,  HPC homogenate,  leech head homogenate. (**a**)—The signal of protons of N-acetyl-D-glucosamine acetyl group of HA chains, (b)—The $^1$H signal of protons at double bond in $\Delta ^{4,5}$-unsaturated glucuronic acid residue.

In this work, the process of HA cleavage by *S. hyalurolyticus* lyase, HPC homogenate, and leech head homogenate was studied. In the case of HPC homogenate and bacterial lyase, two peaks were observed at 2.032 and 2.074 ppm (Fig. 6a). The HA hydrolysis by leech head homogenate results in the appearance of these two peaks roughly from 2.032 and 2.059 ppm. As to the 2.059 ppm peak, it is probably due to the location of N-acetyl-D-glucosamine at the non-reduced end of the HA chains in the case of hydrolysis by leech hyaluronidase, which cleaves the HA by the $\beta$-(1$\rightarrow$3)-glycosidic bond.

The peak area “end protons” shows the total concentration of all formed chains. Thus, using the NMR spectra of the last time points of the experiments, the average number of dimers in the HA chains formed during hydrolysis was calculated. For the reaction mixture with HPC homogenate, the average number of dimers is close to the mixture with *S. hyalurolyticus* lyase (2.62 and 2.59, respectively), for leech head homogenate it is 3.95. In addition, the proton signal ratio of the “end protons” to “inside protons” in reaction mixtures with HPC homogenate and bacterial lyase practically does not differ during the entire time of observation of the course of reactions (Supplementary Fig. 7). This once again demonstrates the similarity of the reaction mechanism of the studied hyaluronidase of the eukaryotic organism to the bacterial one.

It is known that the chemical shift of the H-4 proton at the double bond of unsaturated oligoglucuronates is in the down-field region^43^. As expected, the $^1$H-NMR spectroscopy analysis of the reaction mixture of HA hydrolysis in the presence of leech head homogenate enzymes did not show signals in this region (Figure 6b). In the case of HA cleavage by *S. hyalurolyticus* lyase and HPC homogenate, a proton signal in the down-field region is detected in the spectrum (5.865 ppm). This confirms that HPC hyaluronidase as well as bacterial lyase cleaves HA by the mechanism of $\beta$-elimination with the formation of a double bond.

### Ion-exchange chromatography of the HA after treatment by HPC homogenate

**Figure 7:**
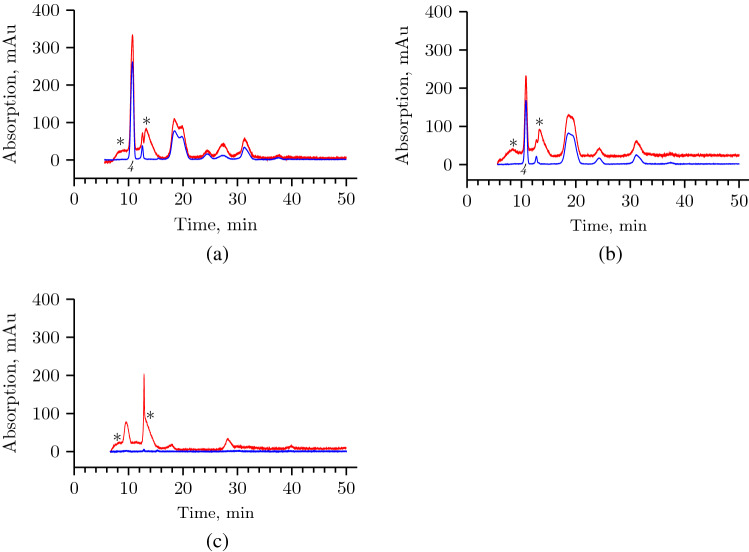
Ion-exchange chromatography of the reaction mixtures containing HA after treatment by: (**a**) *S. hyalurolyticus* lyase, (**b**) HPC homogenate, (**c**) leech head homogenate.  UV absorbance at 210 nm,  UV absorbance at 232 nm.

We carried out ion-exchange chromatography of the reaction mixture for HA cleavage using *S. hyalurolyticus* lyase, HPC homogenate, and leech head homogenate (samples from the end point of the experiment of Supplementary Fig. 7). The absorption at 210 nm detected the cleavage products in all three reaction mixtures (Fig. 7). The absorption at 232 nm was used to detect products containing double bonds in $\Delta ^{4,5}$-unsaturated glucuronic acid residue of oligosaccharides. As expected, absorption at 232 nm was observed in reaction mixtures with *S. hyalurolyticus* lyase and HPC homogenate, and was not observed in the case of reaction products after treatment with leech head homogenate. At the same time, the results showed that the products of HA degradation obtained using red king crab hyaluronidase are almost identical to the products after HA treatment with bacterial lyase detected both at 210 nm and at 232 nm. As is known, the final product of HA cleavage by *S. hyalurolyticus* lyase is the unsaturated tetrasaccharide^23,28,29^. Thus, the final product of HA cleavage by the HPC homogenate is also the unsaturated tetrasaccharide. The difference between the reaction products of these two enzymes is the presence of an additional peak in the case of lyase in the region of 26–28 min of elution. Small absorption peaks at 210 nm to the left and right of the main one (marked with an asterisk) are low molecular weight components of the substrate solution (Supplementary Fig. 8). In the case of leech hyaluronidase, the final product of hydrolysis is tetrasaccharide^25–27^. However, on the chromatogram, the peak corresponding to it, as well as other peaks, is slightly shifted relative to the rest of the chromatogram profiles (Fig. 7(c)). This is probably due to a change in the mobility of oligosaccharides with N-acetyl-D-glucosamine at the non-reduced end and D-glucuronic acid at the reduced end (due to hydrolysis by leech hyaluronidase at the $\beta$-1,3-glycosidic bond HA). The results of ion-exchange chromatography showed that the products of HA degradation obtained using red king crab hyaluronidase are almost identical to the products after HA treatment with bacterial lyase. Unsaturated tetrasaccharide is the final product of the cleavage reaction by red king crab hyaluronidase.

### Substrate specificity: cleavage chondroitin sulfate

Many hyaluronidases show broad specificity and are able to cleave chondroitin sulfate (CS) (Table 1). Using the method of zymography, we showed that the hyaluronidase of the red king crab is able to cleave chondroitin sulfate (Supplementary Fig. 9). However, compared to HA, CS cleavage is much slower, which was shown using the Morgan-Elson reaction (Supplementary Fig. 10) and *in situ*-monitoring of the double bond formation during the $\beta$-elimination reaction of CS (Supplementary Fig. 11). The same insignificant level of CS cleavage was shown by NMR analysis of the reaction mixture containing HPC homogenate (Supplementary Fig. 12). Thus, it can be argued that red king crab hyaluronidase shows slight CSase activity in comparison with HA degradation.

## Discussion

In this paper, we describe for the first time the hyaluronidase activity of a representative of the large group of crustaceans (Decapod order, Anomura Infraorder). At the moment, in the classification of hyaluronidases, it is considered that “certain crustacean” hyaluronidases cleave HA by $\beta$-(1$\rightarrow$3)–glycosidic bonds^7,8,16,44^. This statement is based on the only experimental work where the antarctic krill *Euphausia superba* (Euphausiacea order) hyaluronidase was studied^18^. As shown in our work, red king crab hyaluronidase cleaves HA by $\beta$-(1$\rightarrow$4)–glycosidic bonds by the mechanism of $\beta$-elimination.

It is interesting to note that in the NMR spectra of the reaction mixture for HA cleavage using bacterial lyase and HPC homogenate, an increase in the minor signal is observed in the region of 2.059 ppm (Supplementary Fig. 13). This signal corresponds to the location of N-acetyl-D-glucosamine at the non-reduced end of the HA chains in the case of hydrolysis by leech hyaluronidase (Fig. 6a). This is probably the product of the minor activity of the *S. hyalurolyticus* lyase, which cleaves the HA by the $\beta$-(1$\rightarrow$3)-glycosidic bond^5,6^. If so, then crab hyaluronidase is also able to show this activity.

The most unexpected result in this work is the detection of HA cleavage by the mechanism of $\beta$-elimination by the red king crab hyaluronidase. Traditionally, this mechanism of cleavage is identified only within the “bacterial” type of hyaluronidases. Thus, firstly, our results show that the variety of classes of crustacean hyaluronidases is much wider than we thought up to this point. Secondly, it can now be argued that the mechanism of $\beta$-elimination of HA occurs not only in bacteria and in some eukaryotic microorganisms, but also in complex multicellular animals. Finally, according to the result of searching for homologues of the identified amino acid sequence of red king crab hyaluronidase, it is also possible to judge the uniqueness of the found hyaluronidase among the known ones.

## Methods

### Materials

Rooster comb HA (Sigma H5338) was used to detect the hyaluronidase activity.

The preparation of the hepatopancreas homogenate of crab (HPC) was obtained as described earlier^19^. In short the homogenate was prepared as follows. Frozen hepatopancreas of the red king crab (*Paralithodes camtschaticus*) was thawing at room temperature in laboratory conditions. Unfrozen hepatopancreas, distilled water and ice were added at a weight ratio of 1:8:2, and the mixture was stirred at a low speed for 60 min. The homogenate was filtered (the pore size of the membrane was 300 kDa) and concentrated (the pore size of the membrane was 5 kDa) using a hollow fiber ultrafiltration module. Ammonium sulfate was added to the homogenate to remove impurities from the obtained preparation. After centrifugation, the precipitate was dissolved in a phosphate buffer and dialyzed in the same buffer. The obtained sample was centrifuged and then passed through a 0.22 $\mu$m filter. The sample was analyzed by Laemmli gel electrophoresis (Supplementary Fig. 1)^45^.

The *S. hyalurolyticus* lyase (Sigma H1136) was used. The hyaluronidase product Lydazum (Samson Med LLC, Russia) was of animal origin and was purchased at a pharmacy.

The leech head homogenate was prepared as previously described^46^ with minor changes. 10 leech speciments *Hirudo medicinalis* (Giruda LLC, Russia) were used to obtain the preparation. The leeches were pre-frozen at -70 ${}^\circ$C, then approximately 40–50% of the leech body including the mouth sucker was used. Next, the leech head parts were placed in a mortar with liquid nitrogen and homogenized with a pestle to a state of mush. 150–200 ml of 0.15 M NaCl was added to the obtained material followed by an overnight extraction at a temperature of 4 ${}^\circ$C. The next day, the solution was centrifuged for 15 min at 1500 g at a temperature of 4 ${}^\circ$C. The supernatant was gradually mixed with ammonium sulfate to a final concentration of 70%. The mixture was then centrifuged again for 15 min at 1500 and 4 ${}^\circ$C, after which the precipitate was dissolved in 5 ml 0.1 M sodium acetate, pH 6, and dialyzed against deionized water overnight at 4 ${}^\circ$C.

### Transcriptome analysis, cloning and sequencing of cDNA

The *de-novo* transcriptome assembly approach was used to search for the sequence of mature crab hyaluronidase mRNA. To assemble the transcripts, we used sequencing data on the Illumina HiSeq2500 platform of adult crabs and separately hepatopancreas (SRA NCBI: SRX5509375; SRX5509376; SRX5509377; SRX5509378) with a total volume of more than 13 GBase. Using the CLC Genomics Workbench (Quiagen) software, transcripts were assembled *de-novo*. Then, based on a sample of known insect hyaluronidase sequences and candidates, a candidate RNA carrying the full-length coding sequence of the gene was identified. Based on the mRNA sequence data, specific primers Hyal_F (ATGCCAAAGTTCCAGTTGAGC) and Hyal_R (TTATTTATCTTTCCCTGTGTGAG) were designed.

For cloning, total RNA from the hepatopancreas tissue of the red king crab was isolated using an ExtractRNA kit (Eurogen, Russia) according to the manufacturer’s protocol. For the synthesis of the first DNA chain, Maxima H Minus First Strand cDNA Synthesis Kit (Thermo Fisher Scientific, USA) and oligo(dT)18 were used. Subsequent amplification was performed using Encyclo polymerase (Eurogen, Russia) and specific primers Hyal_F and Hyal_F. Amplicons were cloned in the pGEM-T Easy plasmid vector (Promega, USA), and the hyaluronidase sequence was confirmed by sequencing.

### Enzymatic activity measurement

The HA solution was preheated at the reaction temperature. Then an enzyme preparation was added to the substrate, the reaction mixture was pipetted and incubated. After that, the minitubes with the reaction mixture were placed in a boiling water bath to stop the reaction. In the case of leech head homogenate, a ratio of enzyme preparation to HA was used equal to 1 (volume) to 8–16 (by weight). The reaction mixtures were incubated either at 42 ${}^\circ$C (for lyase *S. hyalurolyticus*), or at 37–38 ${}^\circ$C (for the rest).

To determine the hyaluronidase activity the Morgan-Elson reaction was used^33,34^. The reaction mixture was cooled to room temperature after thermal inactivation. A solution of potassium tetraborate was added to the mixture at a final concentration of 142 mM. After that, the samples were immediately placed onto a boiling water bath for 3 min. Then the samples were cooled again to room temperature, and an Ehrlich reagent containing 0.67 M *p*-dimethylaminobenzaldehyde dissolved in acetic acid and containing 12% hydrochloric acid was added to the samples. After adding the Ehrlich reagent, the mixture was placed in a thermostat and incubated for 20 min at 38 ${}^\circ$C. After that, the absorption was measured at a wavelength of 540 nm on a Multiscan Fc (Thermo Fisher Scientific) plate reader.

Turbidimetry of reaction mixtures of HA hydrolysis using leech head homogenate was performed as described earlier^19^.

The HA $\beta$-elimination reaction was detected by measuring the absorption at a wavelength of 232 nm every 10 s of incubation of the reaction on a thermostated spectrophotometer Varian Cary 100 Bio Spectrophotometer. A solution of HA (150 $\mu$L, 2 mg/ml or 1 mg/ml HA, in phosphate buffer, pH 6, containing 162 mM sodium hydrogen phosphate) was placed in a cuvette and heated for 5 min at the reaction temperature. Next, an enzymatic preparation was added and vigorously mixed. HA in phosphate buffer without the addition of an enzyme preparation was used as a reference solution.

Zymography of hyaluronidases was performed in 10% polyacrylamide gel under denaturing conditions as described earlier with minor changes^47^. Before application, the sample was mixed with a loading buffer (62.5 mM Tris, 100 mM DTT, 0.02% bromophenol blue, 10% glycerin, 0.2% SDS) in a ratio of 1 to 5 by volume, respectively. 2.5 $\mu$g of total protein was applied to the well. The stacking 3.75% polyacrylamide gel contained 0.5 M Tris-HCl (pH 6.8), 1% SDS. 0.05% ammonium persulfate and 0.01% TEMED were used for polymerization. The separating 10% gel contained 1.5 M Tris-HCl (pH 8.8), 1% SDS, 0.2 mg/ml of HA (Sigma H5338) or chondroitin sulfate (Sigma C4384) dissolved in bidistillate, as a substrate. 0.05% ammonium persulfate and 0.01% TEMED were used for polymerization. Electrophoretic separation was performed in a Tris-HCl-Glycine-SDS buffer containing 0.25 M Tris-HCl, 0.192 M glycine, 0.1% SDS, pH 9, at 4 ${}^\circ$C, 50 mA, and 125 V for 4.5 h. After separation, the gel was washed with deionized water, then placed in a buffer containing 0.05 M HEPES and 3% Triton X100 for 1 h to remove SDS from the gel. After that, they were washed with deionized water and rinsed twice in a buffer containing 0.05 M HEPES and 0.15 M NaCl. Next, the gel was placed in an incubation buffer containing 0.15 M NaCl and 0.1 M HCOONa, pH 3.7 (adjusted with HCOOH) at 37 ${}^\circ$C, for 16 h. After that, the gel was stained for 1 h in 1% alcyan blue, dissolved in 3% acetic acid. The gel was washed with a mixture of 7% acetic acid and 10% ethyl alcohol in two shifts of 1 h each.

### NMR spectroscopy of HA cleavage products

One-dimensional (1D) $^1$H-NMR spectra were acquired with a Bruker Avance III 600 spectrometer (The Core Facilities Centre of Institute of Theoretical and Experimental Biophysics of the RAS) operating at a frequency of 600 MHz (1H) and a probe temperature of 311 K or 315 K as described earlier^19^.

### Ion-exchange chromatography

The products of hyaluronan cleavage were analyzed by ion-exchange chromatography on Mono Q 5/50 GL (Amersham Biosciences) column attached to the ProStar HPLC chromatographic system (Varian). Flow rate was 0.6 ml/min. Mobile phase A: 20 mM Tris-HCl, pH 7.4, mobile phase B: 20 mM Tris-HCl, pH 7.4, 1 M NaCl. Aliquots 50 $\mu$l were withdrawn from reaction mixture, diluted 10 fold with the Buffer A and applied to the column followed by the gradient elution according to the program: 4 min—0% B, 6 min—4% B, 59 min—14% B, 66 min—100% B. Absorbance spectra within range of 190–340 nm were collected using photodiode array detector and chromatograms at 210 nm and 232 nm were extracted for presentation.

## Supplementary Information

Supplementary Information.
