# Supplementary material for: β-elimination of hyaluronate by red king crab hyaluronidase
Source: Sci Rep. 2021 Nov 19;11:22600. doi: 10.1038/s41598-021-01890-3 (PMC8604925; doi:10.1038/s41598-021-01890-3)
Supplement: Supplementary file 1 — Supplementary Information. [file 41598_2021_1890_MOESM1_ESM.pdf]

β-elimination of hyaluronate by red king crab hyaluronidase

Dmitrii Sliadovskii[1], Tatyana Ponomareva[1], Maxim Molchanov[2], Irina Pozdnyakova-Filatova[3], Maria Timchenko[1], Victor Marchenkov[4], Oleg Gusev[5,6], and Evgeny Sogorin[1,*]

[1]Federal Research Center “Pushchino Scientific Center for Biological Research of the RAS”, Pushchino, 142290, Russia
[2]Institute of Theoretical and Experimental Biophysics of the RAS, Pushchino, 142290, Russia
[3]Federal Research Center “Pushchino Scientific Center for Biological Research of the RAS”, G.K. Skryabin Institute of Biochemistry and Physiology of Microorganisms, Pushchino, 142290, Russia
[4]Institute of Protein Research RAS, Pushchino, 142290, Russia
[5]Extreme Biology Laboratory, Kazan Federal University, Kazan, 420012, Russia
[6]Department of Regulatory Transcriptomics for Medical Genetic Diagnostics, Juntendo University, Tokyo, 113-8421, Japan
[*]evgenysogorin@gmail.com

ABSTRACT

Crustacean hyaluronidases are poorly understood both in terms of their enzymatic properties and in terms of their structural features. In this work, we show that the hepatopancreas homogenate of the red king crab has a hyaluronidase activity that is an order of magnitude higher than its commercial counterpart. Zymography revealed that the molecular weight of a protein with hyalorunidase activity is 40–50 kDa. Analysis of the hepatopancreas transcriptome and results of cloning and sequencing of cDNA revealed a hyaluronidase sequence with an expected molecular weight of 42.5 kDa. Further analysis showed that hyaluronat enzymatic cleavage follows the β-elimination mechanism, which is well known for bacterial hyaluronidases. The results of ion-exchange chromatography showed that the final product of hyaluronate degradation is unsaturated tetrasaccharide. Thus, we identified a new hyaluronidase of higher eukaryotes, which is not integrated into the modern classification of hyaluronidases.

Supplementary

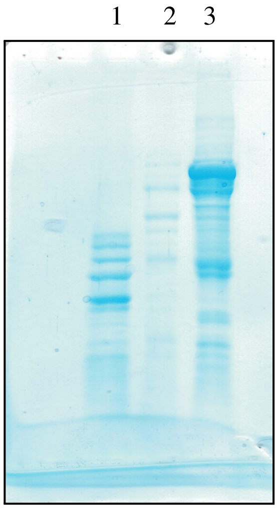

Figure 1. SDS-PAGE electrophoresis of: 1, hepatopancreas homogenate of red king crab (HPC homogenat); 2, Liporase (KORU Pharmaceuticals Co., Ltd, South Korea; was purchased at a pharmacy); 3, Lydazum (Samson Med LLC, Russia; was purchased at a pharmacy).

MPKFQLSQVCLCVVLWSLLSVTSGFNVYWNVPSYLCSKFGIHVNVSKFGIVQN

ANDRFYGDKVNIFYKHGLFPTLNRDGSVRANGGIPQKGNLTAHISTFTHRVKA

QLPHNYTGLAVLDFESYYPSFDMSPPEYRDASRSWVSSLHPKWPGWKVEDEARR

TFNNSAREFFQVLLQVGRELCPGAQWGYYHYPYCHNYQPSITHCQPPVQDHNDA

SLWLIQDSGALYPSIYISENSGWSPRARRLQARVRLGEAVRMAYVAHTPHTPIL

PYFWYRYHDSKERLSSLDLVNTLGLVRMMGLAGAVVWGSSSDLASHDQCLQFKS

YAEDKLGPLVRYLQELPLSSLPRLLRSHRRLRSFVTSALTTSPSHTGKDK*

Figure 2. Amino acid sequence of red king crab hyaluronidase

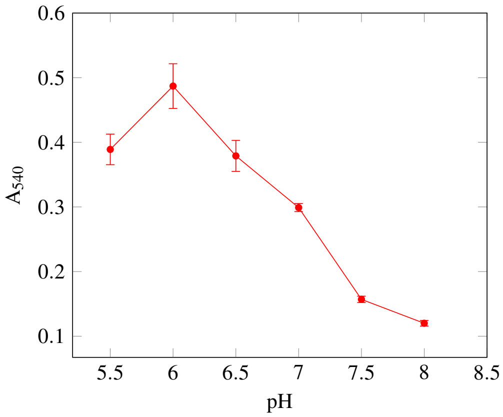

(a)

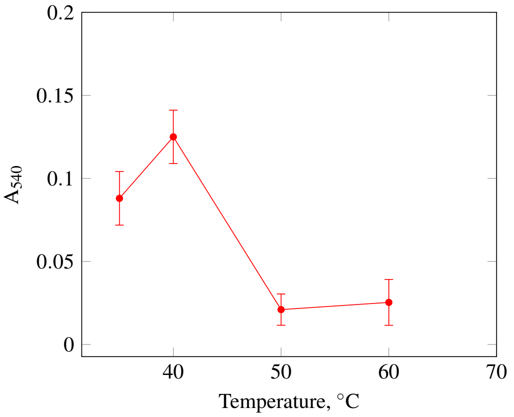

(b)

Figure 3. Cleavage of hyaluronate (2 mg/ml) by HPC homogenate (detection by the Morgan-Elson reaction): (a) in different values of the pH of the reaction mixture (38 °C of reaction mixture, 20 min of incubation time), (b) at different reaction temperatures (pH 6, 20 min of incubation time). The standard deviation calculated by three points is indicated.

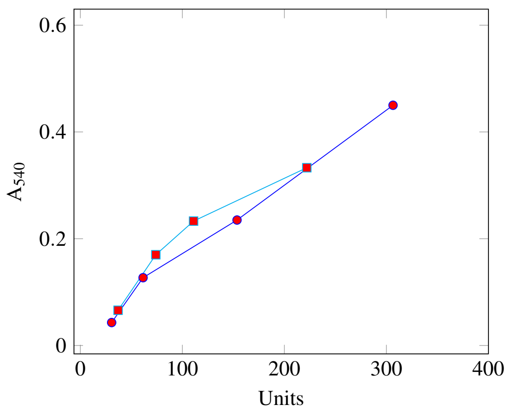

Figure 4. The hyaluronidase activity unit titration in the reaction mixture of the HPC homogenate (—●—) and the commercial drug of the *S. hyalurolyticus* lyase (—■—) (Sigma H1136). Detection was carried out by the Morgan-Elson reaction (2 mg/ml HA in the phosphate buffer, pH 6, and at 38 °C during 20 min of the reaction).

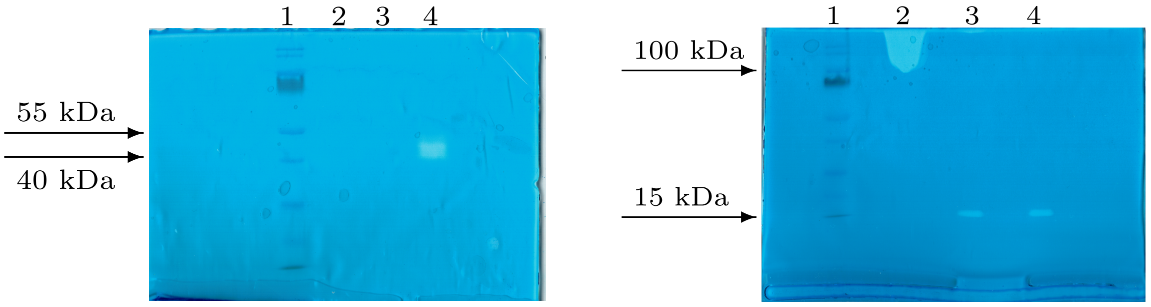

(a) (b)

Figure 5. Zymogram in polyacrylamide gel containing HA as a substrate: (a)—HPC homogenate (1—protein marker, 2—homogenate after heating in the loading buffer, 3—homogenate after heating in the loading buffer without DTT, 4—homogenate in buffer with DTT without warming up), (b)—*S. hyalurolyticus* lyase (1—protein marker, 2—after warming up in buffer without DTT, 3—after warming up in buffer with DTT, 4—hyaluronidase in buffer with DTT without warming up). Protein marker PageRuler Prestained Protein Ladder: 180, 130, 100, 70, 55, 40, 35, 25, 15, 10 kDa. The samples were being heated in the loading buffer for 5 minutes at 95 °C.

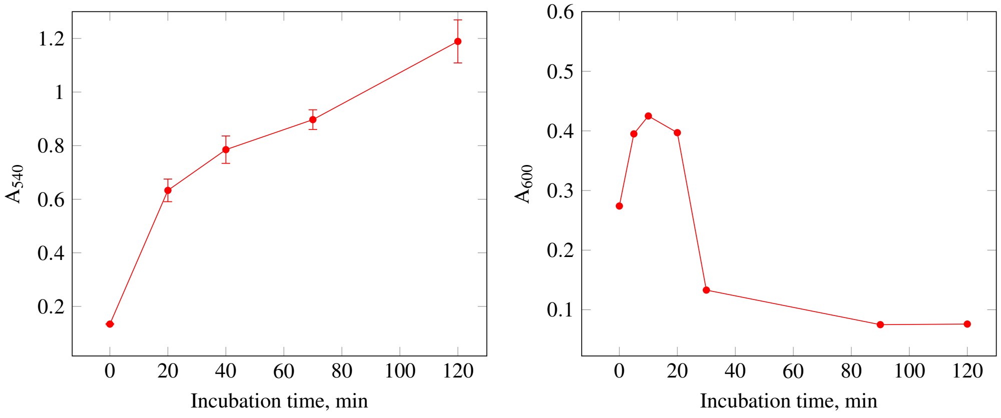

(a) (b)

Figure 6. Hydrolysis of hyaluronate: (a) using the Lydazum drug (detection by the Morgan-Elson reaction, the standard deviation calculated by three points is indicated), (b) using leech head homogenate (turbidimetric analysis).

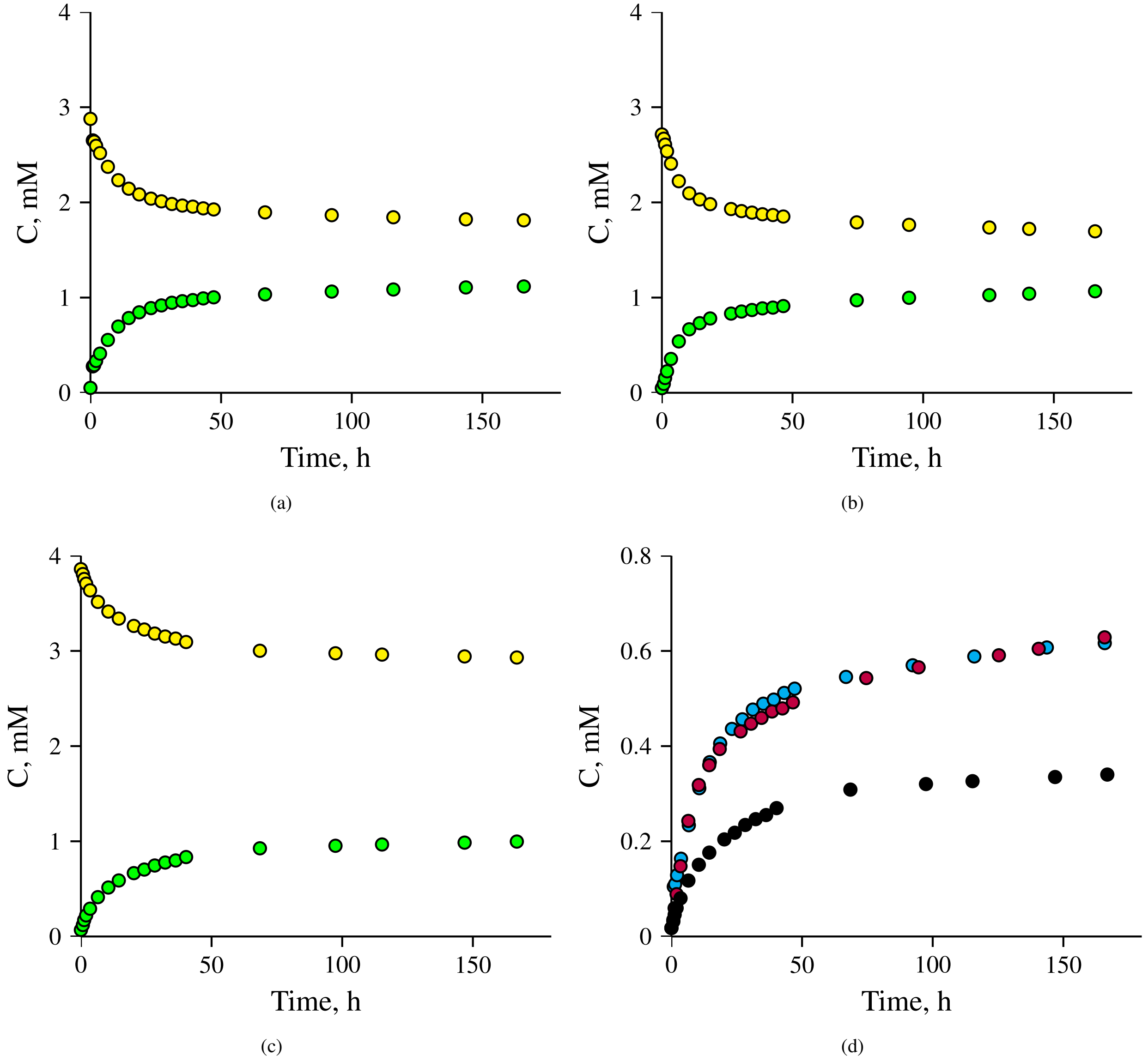

Figure 7. Kinetics of ^{1}H signal accumulation of N-acetyl-D-glucosamine acetyl group of HA chains (integral under peaks) during treatment by: (a) HPC homogenate (), (b) *S. hyalurolyticus* lyase, (c) leech head homogenate. The mark presents the proton signals of the N-acetyl-D-glucosamine acetyl group inside the HA chain ("inside protons"), the mark represents the proton signals of the N-acetyl-D-glucosamine acetyl groups, which is formed at the ends of the HA fragments ("end protons") during cleavage. (d) Proton signal ratio of the "end protons" to "inside protons", which are detected during HA cleavage by HPC homogenate (), *S. hyalurolyticus* lyase (), leech head homogenate (). 2 mg/ml HA in the phosphate buffer, pH 6, 38 °C. The molar concentration of HA was estimated from its disaccharide units as described earlier[1].

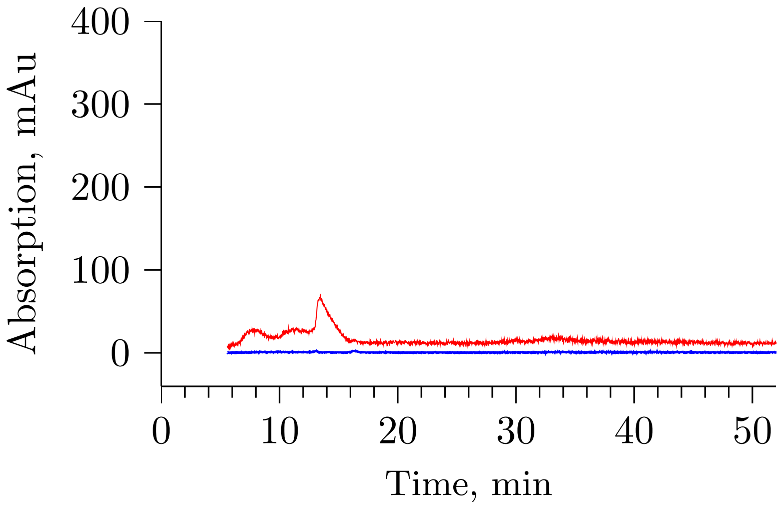

Figure 8. Ion-exchange chromatography of the HA solution before treatment: — UV absorbance at 210 nm, — UV absorbance at 232 nm.

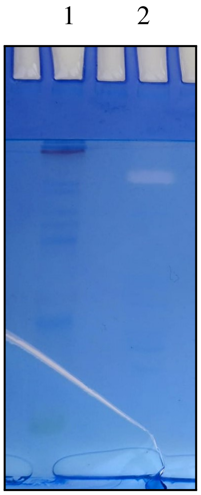

Figure 9. Zymogram in polyacrylamide gel containing chondroitin sulfate as a substrate: 1—protein marker, 2—HPC homogenate. Protein marker PageRuler Prestained Protein Ladder: 180, 130, 100, 70, 55, 40, 35, 25, 15, 10 kDa.

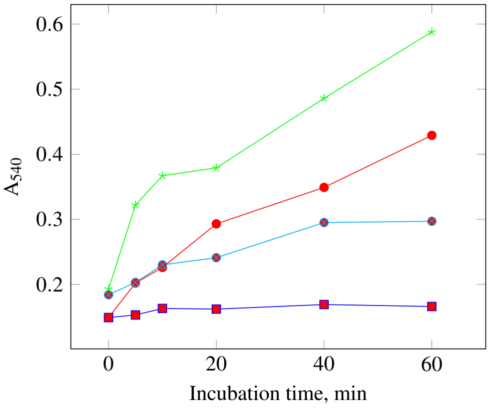

Figure 10. Hydrolysis of chondroitin sulfate (2 mg/ml in the phosphate buffer, pH 6, 37 °C) using the Lydazum drug or HPC homogenate (detection by the Morgan-Elson reaction): Lydazum (64 units), HPC homogenate (64 units), HPC homogenate (307 units), HPC homogenate (1228 units). The hyaluronidase activity in the reaction mixture is indicated in brackets (the commercial drug Lydazum was used as a standard control to define this hyaluronidase activity).

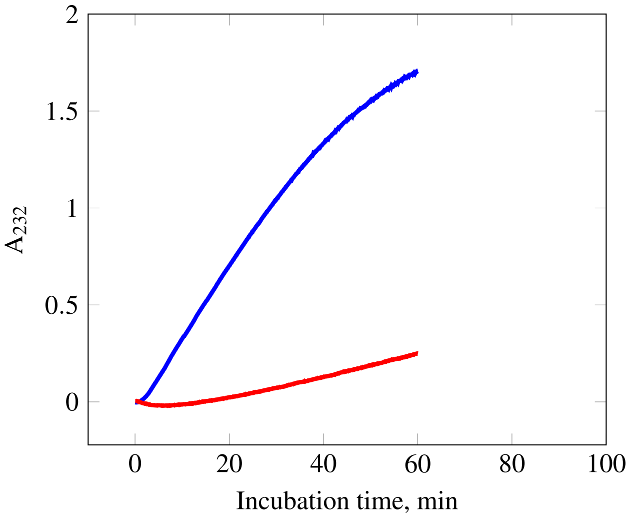

Figure 11. *In situ*-monitoring of the double bond formation during the β-elimination reaction of hyaluronate ( ) or chondroitin sulfate ( ) by HPC homogenate (2 mg/ml in the phosphate buffer, pH 6, 37 °C).

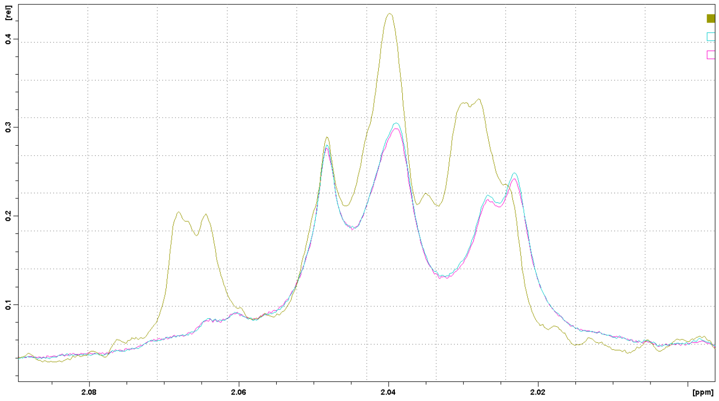

Figure 12. Changes in the ^{1}H signal of the N-acetyl-D-glucosamine acetyl group of chondroitin sulfate (CS) chains in ^{1}H-NMR spectra during treatment by HPC homogenate: 47 min, 77 min, 70 h of the reaction. 2 mg/ml CS in the phosphate buffer, pH 6, 38 °C.

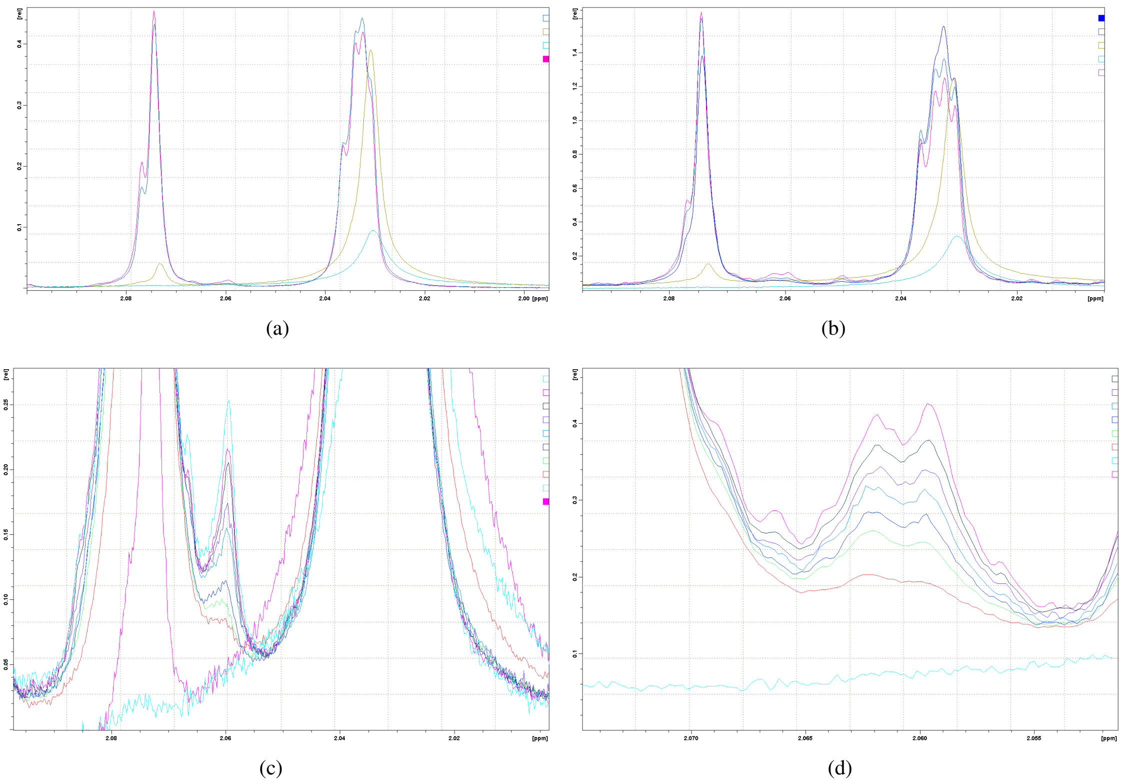

Figure 13. Changes in the ^{1}H signal of the N-acetyl-D-glucosamine acetyl group of HA chains in ^{1}H-NMR spectra during treatment by (a) *S. hyalurolyticus* lyase: — HA before the treatment, — 44 min of the reaction, — 75 h, — 166 h; (b) during treatment by HPC homogenate: — HA before the treatment, — 55 min of the reaction, — 23.2 h, — 67 h, — 166 h of the reaction. 2 mg/ml HA in the phosphate buffer, pH 6, 38 °C. (c)— scaled signal area around 2.059 ppm shown in (a), (d)—scaled signal area around 2.059 ppm shown in (b).

A Methods

Turbidimetric analysis (Supplementary Figure 6(b)) was conducted according to previously developed protocols, with minor changes[2–4]. The hyaluronate (HA, Rooster comb HA, Sigma H5338) was diluted by phosphate buffer (160 mM disodium phosphate and 39 mM sodium chloride; adjusted to pH 6 with hydrochloric acid) to a concentration of 2 mg/mL HA. An aliquot of the hyaluronidase solution (0.1 mL) was added to 0.4 mL of the HA solution, and the reaction mixture was thoroughly mixed and incubated at 38 °C. At certain time points during incubation, 50 μL aliquots were taken and then immediately mixed with 250 μL of acidic albumin (24 mM sodium acetate, 79 mM acetic acid, 50 mM sodium chloride, 1% albumin, pH 3.5). The mixture was thoroughly mixed and incubated at room temperature for 50 min. The absorbance was then measured at 600 nm on Multiscan Fc (Thermo Fisher Scientific) plate reader.

A.1 Note

Turbidimetric analysis is based on two properties of high molecular weight HA: it binds to albumin in acidic conditions, and it forms aggregates. Light scattering by the aggregates was determined by measuring the optical density of the sample. As soon as the high molecular weight forms of HA are transformed into low molecular weight forms (as a result of hydrolysis), aggregates can no longer form. Thus, a decrease in the absorption at 600 nm indicates the hydrolysis of HA at the reaction time. The reason for the increase of the absorption during the first 20 minutes of incubation remains unclear. Probably, there is an optimal size of a high-molecular HA to efficiently form aggregates. After 20 minutes, the expected decrease in the formation of aggregates is observed, which indicates the hyaluronidase activity of leech head homogenate.

References

1. Ponomareva, T. *et al.* The effect of hepatopancreas homogenate of the red king crab on ha-based filler. *PeerJ* **8**, e8579 (2020).

2. Dorfman, A. & Ott, M. L. A turbidimetric method for the assay of hyaluronidase. *J. biol. Chem* **172**, 367–375 (1948).

3. Rapport, M. M., Meyer, K. & Linker, A. Correlation of reductimetric and turbidimetric methods for hyaluronidase assay. *J. Biol. Chem.* **186**, 615–623 (1950).

4. Tam, Y.-C. & Chan, E. Modifications enhancing reproducibility and sensitivity in the turbidimetric assay of hyaluronidase. *J. Microbiol. Methods* **1**, 255–266 (1983).
